# A hybrid cloud read aligner based on MinHash and kmer voting that preserves privacy

**DOI:** 10.1038/ncomms15311

**Published:** 2017-05-16

**Authors:** Victoria Popic, Serafim Batzoglou

**Affiliations:** 1 Department of Computer Science, Stanford University, Stanford, California 94305, USA

## Abstract

Low-cost clouds can alleviate the compute and storage burden of the genome sequencing data explosion. However, moving personal genome data analysis to the cloud can raise serious privacy concerns. Here, we devise a method named Balaur, a privacy preserving read mapper for hybrid clouds based on locality sensitive hashing and kmer voting. Balaur can securely outsource a substantial fraction of the computation to the public cloud, while being highly competitive in accuracy and speed with non-private state-of-the-art read aligners on short read data. We also show that the method is significantly faster than the state of the art in long read mapping. Therefore, Balaur can enable institutions handling massive genomic data sets to shift part of their analysis to the cloud without sacrificing accuracy or exposing sensitive information to an untrusted third party.

## Abstract (web summary)

Outsourcing computation for genomic data processing offers the ability to allocate massive computing power and storage on demand. Here, Popic and Batzoglou develop a hybrid cloud aligner for sequence read mapping that preserves privacy with competitive accuracy and speed.

Recent sequencing technology breakthroughs have resulted in a dramatic increase in the amount of available sequencing data, enabling major scientific advances in biology and medicine. At the same time, the compute and storage demands associated with processing genomic data have also substantially increased and now often outmatch the in-house compute capabilities of many research institutions. Outsourcing computation to commercial low-cost clouds (for example, Amazon Elastic Compute Cloud, Azure, Google Cloud), which offer the ability to allocate massive compute power and storage on demand, provides a convenient and cost-effective solution to this problem. However, exposing genomic data to an untrusted third party also raises serious privacy concerns since genomic data carry extremely sensitive personal information about its owner, such as ethnicity, ancestry and susceptibility to certain diseases. A recent review^1^ describes the growing concern over the ability to protect personal genetic privacy. As summarized by the review, the privacy of genetic information is currently a demand of many regulatory statutes in the United States and the European Union and a major determinant of whether individuals are willing to participate in scientific studies. While deidentification and anonymization techniques have been suggested as solutions for this problem, it has been shown that such techniques cannot reliably prevent the identification of an individual from genomic data^1^,^2^,^3^,^4^. For example, numerous identity-tracing attacks have been demonstrated using quasi-identifiers, including demographic metadata^5^, pedigree structures^6^ and genealogical data^7^. The vulnerabilities of such approaches motivate the need for cryptographic techniques to process and store genomic information.

Read alignment is a critical and computationally intensive first step of most genomic data analysis pipelines. While tremendous effort^8^,^9^,^10^ has been dedicated to this problem, few approaches have addressed outsourcing this computation securely to an untrusted party. The few secure solutions that exist either do not scale to whole genome sequencing data sets^11^,^12^,^13^ or are not competitive with the state of the art in read mapping^14^. In particular, the protocol^11^ for computing edit distances using homomorphic encryption^15^ requires 5 min on a single pair of 25 bp sequences^12^, and the approach^13^ using secure multiparty computations, while more efficient, still takes 4 s for a pair of 100 bp sequences. Recently, Chen *et al*.^14^ proposed a secure seed-and-extend read mapping algorithm on hybrid clouds that splits the computation such that the public cloud finds the exact seed matches using encrypted seeds and the private cloud extends the seed matches using unencrypted data. With this approach, mapping 10 million 100 bp reads takes 370 CPU hours on the public cloud, an additional 2 h on the private cloud and 6.8 TB to store the reference index. This time requirement is not competitive with state-of-the-art aligners, given that time spent on the private client alone is higher than the total runtime of standard aligners. For instance, Bowtie2 requires 1 h for the same data with a peak memory footprint of just 3.24 GB (ref. 10).

In this work we present Balaur, an efficient privacy preserving read mapping technique for hybrid clouds based on locality sensitive hashing and kmer voting. Balaur securely outsources 50–70% of the read mapping task to the public cloud, while being highly competitive with existing state-of-the-art aligners in speed and accuracy. Therefore, in a hybrid cloud system with a fast network, Balaur can result in substantial savings of private cloud resources with respect to standard nonsecure tools that must entirely run on the private cloud.

## Results

### Overview of the Balaur framework

**Figure 1:**
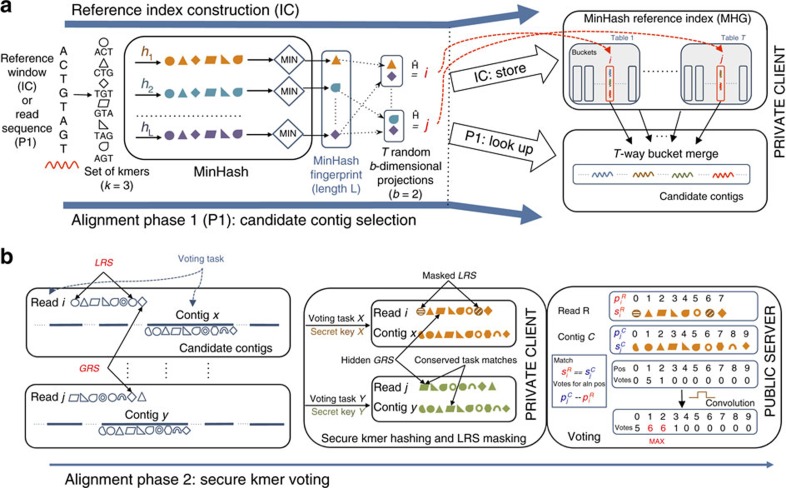
Overview of Balaur. (**a**) Illustration of the MHG index construction (IC) and candidate contig selection (P1) protocols for a given reference window or read sequence, respectively. Given the sequence MinHash fingerprint (computed using *L* random hash functions *h*_1_…*h*_*L*_), we store the sequence in *T* MHG buckets according to the values of the *T* fingerprint projections (during IC); or we look up and merge the contents of the corresponding *T* buckets (during P1). (**b**) Illustration of the kmer voting protocol. The read and candidate contig sequences are decomposed into a set of kmers; initial LRS and GRS repeats are illustrated using identical shapes; the results of cryptographically hashing the kmers using different keys is shown using different colours; the results of masking LRS is shown using different stripe patterns. For simplicity, the voting results are shown without LRS neighbour masking and kmer position binning.

At a high level, Balaur can be summarized in the following two phases (Fig. 1): (1) fast identification of a few candidate alignment positions in the genome using the locality sensitive hashing^16^ (LSH) scheme MinHash^17^ (on the secure private client) and (2) evaluation of each candidate using secure kmer voting (on the untrusted public cloud).

We leverage LSH and the MinHash in phase 1 by formulating the task of finding the candidate alignment positions as nearest neighbour search under the Jaccard set similarity criterion, searching for reference sequences that are most similar to the reads. To rapidly find such sequences, we first pre-compute the MinHash *fingerprints* of reference genome windows and store them in our MinHash reference genome index (MHG) data structure (Fig. 1a, index construction). In particular, we store each window in *T* different buckets of the index, each corresponding to a *b*-dimensional projection of its fingerprint.

**Figure 2:**
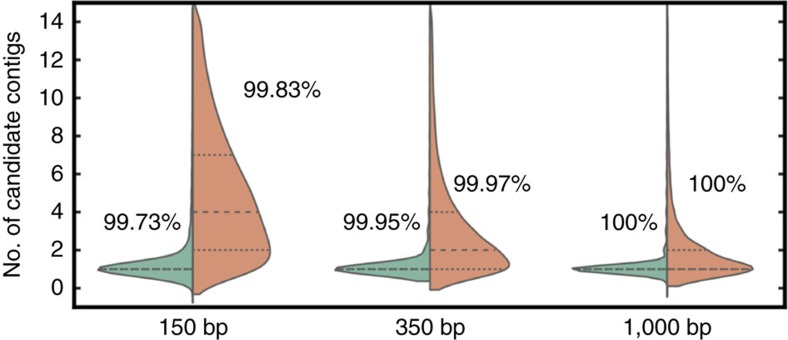
Distribution of the number of candidate contigs (cropped) selected per read from the MHG for varying read lengths. The data sets used consist of 100 K reads simulated from GRCh37 with wgsim^13^ using default parameters and a sequencing error rate of 1%. The percentage of reads for which the true contig (that is, the contig covering the true alignment position) was selected is shown on top of each distribution. For each read length, two distributions are shown according to the setting of the *b*_min_hits_ parameter that represents the minimum number of MHG buckets that must contain the contig for it to be selected as a candidate: *b*_min_hits_=1, all contigs in the buckets (orange); *b*_min_hits_=2, contigs present in at least two buckets (green).

To scale the MHG construction to the full human genome, we developed a ‘rolling' MinHash optimization technique and a memory reduction scheme that compresses consecutive genome windows that hashed to the same bucket into single *contig* entries defined by a start genome position and length. Given the MHG, phase 1 of our algorithm then consists of computing the MinHash fingerprint of each read and merging the contents of its corresponding *T* index buckets into a set of candidate reference contigs (Fig. 1a, candidate contig selection (P1)). As can be seen in Fig. 2, the MHG allows us to select <10 candidate contigs for most reads in our data sets. This high selectivity is crucial for our alignment algorithm, since it significantly decreases the overheads associated with transferring the selected contigs for secure voting in phase 2.

In phase 2 of the algorithm (Fig. 1b), we estimate the read alignment position and score for each candidate contig using kmer voting. Given a *voting task* (a read and candidate contig pair), each kmer in the read matching a kmer in the contig *votes* for the alignment position given by the difference in the kmer displacements. Since we expect some votes to be split across neighbouring positions due to indels, we perform a simple convolution step to collect neighbouring votes. The position with most votes across all the contigs is selected as the final alignment position of the read; note that all voting tasks can be processed in parallel.

Our goal is to outsource phase 2 securely to the cloud. We assume the ‘honest-but-curious' adversary cloud model that follows the read alignment protocol correctly but might try to conduct inferences from the inputs and background knowledge, such as the reference genome. More specifically, if the attacker can detect *local* or *global* repeat structures, kmer collisions within (local repeat structures (LRS)) or across (global repeat structures (GRS)) each read or contig, respectively, it can devise a variety of statistical attacks using this information (see Methods for details). A key property of our voting protocol is that it does not require the comparison of kmers across voting tasks, since we only need to detect kmer matches between a read and its candidate contig to count votes. This allows us to securely hash the kmers using different secret keys for each voting task, thus avoiding GRS leaks. To avoid LRS leaks, we hide local repeats by masking them along with their neighbours. Finally, to compute the position voted for by each kmer match, we reveal the partial ordering of the kmers inside the read and contig, shuffling kmers falling into different bins, such that the position of each kmer is ambiguous within *β* positions and only the relative positions of the bins are known. We describe our voting scheme in detail and analyse its security guarantees in the Methods section. We address and prevent all attacks known to us that could use the information available in the cloud; however, we do not rigorously prove that an attack is impossible.

### Performance evaluation on simulated data

To assess the accuracy of our approach we simulated data sets of 150 and 350 bp long reads from the human genome reference GRCh37 using the wgsim program^18^ with default parameters and sequencing base error rates of 1 and 2%. We compare Balaur with several popular and efficient state-of-the-art nonprivate read aligners: BWA-MEM^8^, Bowtie2 (ref. 10), CUSHAW3 (ref. 19), ALFALFA^20^ and SOAP2 (ref. 21). All the experiments were performed on a single 2.67 GHz Intel Xeon X5550 processor. The accuracy results in a distributed setting will remain the same, while some additional costs will be incurred due to the client–cloud communication. Runtime is reported for single-threaded execution (unless noted otherwise). We do not incorporate initialization costs (for example, the allocation of kmer transfer buffers and MHG loading) into the reported Balaur runtime. We report the percentage of reads aligned with a mapping quality of ≥10 (Q10) and the percentage of incorrect alignments out of the Q10 mappings (Err). We considered a read to be mapped correctly if its reported position was within 20 bp of the true position. We describe in detail the parameters used to execute Balaur and all other benchmarked aligners in Supplementary Notes 1 and 3.

**Figure 3:**
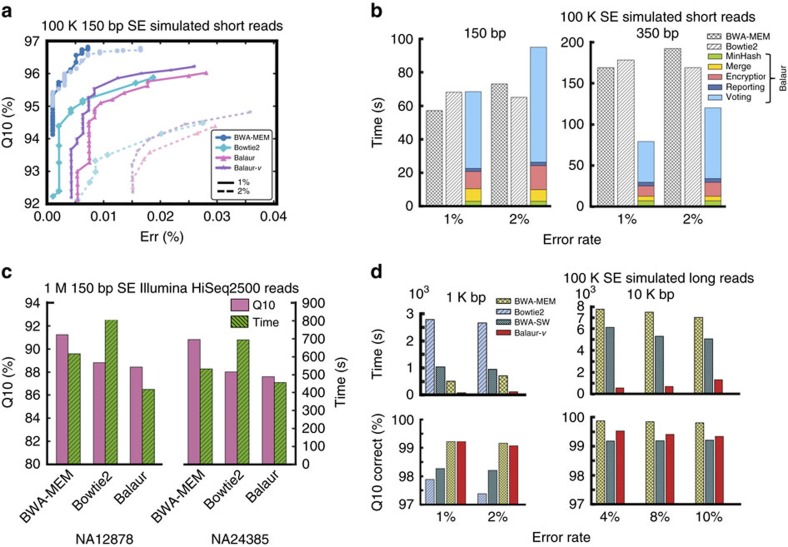
Performance comparison of Balaur and other read mapping tools. (**a**) Accuracy results on simulated 150 bp long reads with 1 and 2% sequencing error rates. (**b**) Runtime comparison and breakdown by alignment phase on 150 and 350 bp simulated reads. The reported ‘encryption' time includes both secure kmer hashing and repeat masking, while the ‘reporting' time represents the post-processing of the voting results on the client (that is, selecting the best alignment positions and SAM output file I/O). (**c**) Evaluation on real read data. (**d**) Evaluation on simulated long read data with varying sequencing error rates showing the runtime and the percentage of Q10 reads that were mapped correctly (within 20 bp of true alignment position).

Figure 3a,b and Supplementary Table 1 show that Balaur performs well in comparison with other aligners, achieving similar high percentages of Q10 mapped reads and low error rates. To quantify the effects of LRS and position binning, Fig. 3a also shows the performance of the nonsecure version of our aligner, Balaur-*v*anilla (which uses a faster noncryptographic hash function, no repeat masking and no position binning). It can be seen that masking and binning do not have a significant impact on the accuracy of the alignment results. Further evaluation of security-related settings and the effect of sampling contig kmers are shown in Supplementary Fig. 1.

### Performance evaluation on real data

Next we assessed the performance of Balaur on real read data. We aligned the following two data sets to the GRCh37 reference: (1) 1 M 150-bp HiSeq2500 reads of the NA12878/HG001 genome and (2) 1M 150-bp reads of the NA24385/HG002 genome (Fig. 3c and Supplementary Table 2). As in simulations, Balaur achieves results similar to the other aligners, with 99% of Balaur Q10 mappings being identical to BWA-MEM mapping results.

**Figure 4:**
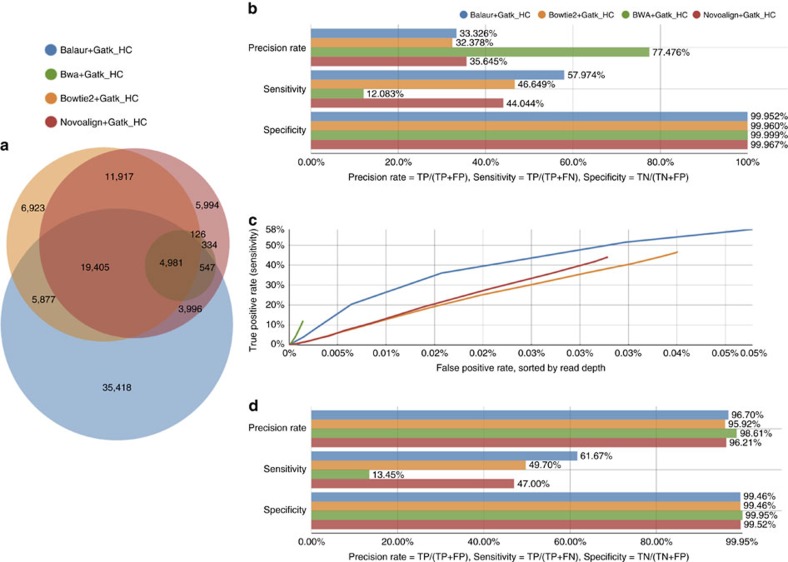
Evaluation of variant calling using the GCAT benchmarking platform. Results are shown for the NA12878 Ion Torrent 225-bp SE exome 30 × data set available through GCAT^22^. Reads were aligned against the UCSC hg19 human genome reference. Balaur variant calling was performed using the GATK HaplotypeCaller pipeline^23^ (see Supplementary Note 2 for details). Comparisons are shown against the Bowtie2, BWA and Novoalign aligners with GATK HaplotypeCaller variant calling reports available through GCAT. All subfigures were generated by GCAT given the VCF file containing the Balaur variant calling results. (**a**) Venn diagram showing the concordance of the results obtained for each aligner. (**b**,**c**) Comparison against the NIST Genome in a Bottle SNP and indel call set (v2.18). (**d**) Comparison against the HumanOmni2.5-8v1 Illumina genotyping array. For more information about the benchmarks please see GCAT^22^.

To validate our results further, we used the GCAT (genome comparison and analytic testing)^22^ benchmarking platform to test variant calling on the provided NA12878 Ion Torrent 225-bp 30 × exome read data set. We performed variant calling using Balaur alignments and the GATK HaplotypeCaller^23^ pipeline as described in Supplementary Note 2. Balaur-based results are highly competitive with other methods evaluated by GCAT (Fig. 4 and Supplementary Table 3).

### Long read mapping

Orthogonally to security guarantees we also explored the effectiveness of Balaur as a long read aligner. We simulated 1K bp and 10K bp read data sets from GRCh37 using wgsim^13^. We used the wgsim default polymorphism parameters and set the base error rate to 1 and 2% for the 1K bp data set and 4, 8, and 10% for the 10K bp reads, respectively. We compare our results with the BWA-MEM, BWA-SW^9^ and ALFALFA^20^ aligners that were designed to support long read alignment. For this experiment we used Balaur-*v*anilla, kmer length of 32 bp and varying sampling rates and MHG index sizes. As shown in Fig. 3d and Supplementary Table 4, Balaur-*v*anilla achieves significant speedups over the other aligners, ranging from 2 to 40 × , while maintaining high accuracy and sensitivity.

### Runtime and communication cost breakdown

Finally, we break down the runtime and bandwidth costs associated with each alignment phase (Fig. 2b and Supplementary Table 5). While our approach cannot transfer all the computation to the cloud, the outsourced voting step accounts for roughly 50–70% of the total runtime and can be efficiently parallelized (Supplementary Fig. 2 shows how our cloud-side computation scales with the number of available processors). We also find that although high, the imposed communication overheads are still practical for current networking speeds (moreover, cloud providers, such as Amazon EC2, do not charge users for data transfer). For example, for the 1 M NA12878 real read experiment, our method requires 24.6 GB bandwidth to ship the voting tasks that would take 197 s to transfer on a fast 1 Gbps link. Furthermore, by overlapping data transfer and computation at the client side, one can hide these communication delays.

## Discussion

Ongoing advances in DNA sequencing technologies have undoubtedly shifted genomics into the realm of big data, such that the costs of processing and storing sequencing data now exceed the costs of generating it. Low-cost cloud computing can alleviate this problem; however, new methods must be developed to practically shift genome analysis to the untrusted cloud without compromising the privacy of the individuals' genomic information.

Here, we presented Balaur, a practical secure read mapping algorithm for hybrid clouds. It splits the computation between the trusted private client and the untrusted cloud using the MinHash algorithm on the client and a secure kmer voting scheme on the cloud. We analysed the security aspects of our approach, describing what information it leaks to the cloud and addressing any adversarial attacks known to us.

While Balaur outsources 50–70% of the task to the cloud, it uses client resources for the rest of the computation, as well as transfers a large amount of data from the client to the cloud. Therefore, methods that can minimize this client-side compute and data transfer or ideally shift the entire computation to the cloud must still be developed. Furthermore, while our work addresses read mapping, privacy-preserving algorithms for the subsequent steps of the genome analysis pipeline (for example, local realignment and variant calling) must also be developed to practically move the entire genome analysis pipeline to the cloud.

## Methods

### MinHash fingerprinting

In this work we hash the reference genome and the reads using LSH, such that the hash values (referred to as fingerprints) of the read and its genome position collide with high probability. In particular, we use the standard Jaccard set similarity coefficient to compare the read and genome sequences, defined as 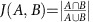 for two sets *A* and *B*, and the min-wise independent permutations (MinHash) LSH scheme to rapidly estimate *J*(*A*, *B*) (see Supplementary Note 4 for background information).

To apply the Jaccard similarity measure, we represent sequences as sets of overlapping kmers (that is, substrings of a given length *k*), filtering out highly repetitive kmers.

Given the set *K*={*s*_0_*, s*_1_*, ..., s*_*n−1*_} of kmers of length *k* of a sequence *S*, its MinHash fingerprint vector **F**=[*f*_0_*, f*_1_*, ..., f*_*L−*1_] is created as follows. First, each kmer in the set is hashed using some hash function *H*, resulting in the set of kmer hash values *K*_*H*_={*H*(*s*_0_), *H*(*s*_1_),..., *H*(*s*_*n*−1_)}. We then apply *L* random universal hash functions of the form *h*_*i*_(*x*)=*a*_*i*_*x*+*b*_*i*_ to each element of *K*_*H*_. Each fingerprint entry *f*_*i*_ is the minimum element in the set under the hash function *h*_*i*_:


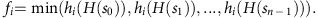


### MinHash reference genome index

By the MinHash property, the number of entries shared by two given fingerprints is equivalent to the similarity of their sets, near-duplicate sequences producing fingerprints with many identical *f*_*i*_ entries. Since pairwise fingerprint comparisons would result in an infeasible running time, we construct an index of the reference genome, the MHG, to find alignment candidates more rapidly.

The MHG consists of *T* hash tables (with *B* buckets per table, such that *B*=2^*M*^) storing locations in the reference genome. We populate the index using LSH as follows. For each window *w* of the genome (of length equal to the read length) and its MinHash fingerprint **F**, we generate *T b*-dimensional projections of **F** (one per hash table). The projections are computed using *T* fixed *b*-dimensional vectors of randomly selected indices into the fingerprint. Let **proj**_**F**_ be the *t*th projection of the fingerprint vector **F** (where *t*<*T*) and **P** the *t*th vector of fingerprint indices, then **proj**_**F**_[*i*]**=F**[**P**[*i*]], for all *i<b*. The hash value *h* of the projection is then computed using a standard multiply-shift hash function initialized with a vector **A** of random odd 64-bit integers, such that *h*=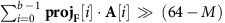. Finally, *w* is placed into bucket *h* of hash table *t*. As a result, each hash table bucket will store all genome locations that share at least *b* fingerprint entries. The index construction is schematically illustrated in Fig. 1a.

Using the fact that consecutive windows of the genome are related, we developed a rolling MinHash technique to compute the fingerprints more efficiently. Supplementary Note 5 describes how the fingerprint of window *w*_*p*+1_ can be computed from that of window *w*_*p*_, where *p* and *p+*1 are consecutive positions in the genome.

There is a clear tradeoff between the number of hash tables *T* and the sensitivity of the algorithm, more hash tables allowing us to identity more potential hits. However, since space consumption scales linearly with *T*, the memory requirement for indexing the full human genome can be too high for large enough *T*. To reduce the memory footprint of the index, we use the following scheme: instead of storing separate genome positions, we store contigs, defined by a position and a length, that represent consecutive genome windows that hashed to the same bucket under the same projection. As a result, the produced indices are significantly smaller in size. The greatest compression is achieved for larger window lengths. For example, for window lengths of 150 bp, 2.83 B genome windows were found to have valid MinHash fingerprints; bucketing them into *T*=78 tables resulted in 6.58 B total entries across all the tables that constitutes a 33.5 × size reduction. For windows of length 1,000 bp, the reduction in size was 245 × . The memory requirements for the full GRCh37 MHG index under the parameters *L*=128, *T*=78, *b*=2 and *M*=18 used in our experiments vary as follows: 74 GB (150 bp), 30 GB (350 bp), 11 GB (1,000 bp) and 1.1 GB (10,000 bp).

We parallelized the index construction algorithm using OpenMP and use TBB C++ scalable allocators to optimize the frequent bucket memory allocations. To eliminate lock contention, each thread maintains local buckets while processing its regions of the reference genome; the per-thread local buckets are then merged upon completion. Given the present scheme, indexing the full reference genome on the Intel Xeon X5550 processor takes 1.3 h using 4 cores for 1,000 bp windows with the parameter setting *L*=128, *T*=78, *b*=2 and *M*=18.

### Candidate contig selection during phase 1 on private client

During the first phase of the alignment process we compute the MinHash fingerprints of each read to identify a small set of candidate alignment positions in the genome that are expected to be similar to the reads under the Jaccard similarity metric. Given the fingerprints, we also calculate the *T b*-dimensional projection hash values to retrieve the genome windows that share the fingerprint entries selected by each projection. Each of these hash values corresponds to a particular bucket in the *T* index hash tables. Thus, we obtain *T* buckets, each containing contigs of windows that share at least *b* entries with the read (we use *b*=2 in our experiments that guarantees that the candidate windows and the read share at least two entries in their fingerprints). This step comprises only a small portion of the total alignment runtime for typical read lengths and is not currently outsourced to the public cloud to avoid the communication costs associated with shipping the read kmers, as well as potential inference attacks.

Intuitively, the more buckets a window shares with the read, the more likely it is to be the correct alignment: a perfect match, for instance, would share all *T* buckets. Therefore, given the contigs from all the buckets, the final step in identifying the best candidate contigs is to count the total number of buckets each contig is present in, *b*_hits_. To do so, our client performs a simple *n*-way merge using a min-priority heap that stores (contig, *b*_id_) tuples of the entries in the buckets (where *b*_id_ uniquely identifies each bucket), relying on the fact that the buckets have been sorted by increasing position during indexing. We use a threshold *b*_min_hits_ to represent the minimum *b*_hits_ required for a contig to be considered a strong candidate, as well as keep track of the highest *b*_hits_ seen so far, *b*_best_hits_. A given contig is passed to the next alignment step if its *b*_hits_ ≥ *b*_min_hits_ and it is within some predefined range from *b*_best_hits_. Figure 2 illustrates the effectiveness of the MinHash index in reducing the number of candidate alignment positions to only a few contigs per read.

### Kmer voting during phase 2 on public server

Given a candidate contig, the second phase of the algorithm is to estimate the displacement *ϕ* that best aligns it to the read, as well as a score that measures the quality of that alignment. We define a voting task to represent a read and contig pair and employ the following voting technique to process each task resulting from phase 1 (each task can be executed in parallel on the cloud).

Let us start by representing a read *R* and a candidate contig *C* as a set of kmers and their associated positions: {...(*s*_*i*_^*R*^, *p*_*i*_^*R*^)...} and {...(*s*_*i*_^*C*^, *p*_*i*_^*C*^)...}, respectively (our implementation currently uses 20 bp overlapping kmers). Assume we have found the correct contig. With no error and only unique kmers, a single match *M*_(*i,j*)_ :=(*s*_*i*_^*R*^==*s*_*i*_^*C*^) would unambiguously yield the correct alignment. Namely: *ϕ*_(*i,j*)_=*p*_*i*_^*C*^−*p*_*i*_^*R*^ . We say that match *M*_(*i,j*)_ votes for the alignment position *ϕ*_(*i,j*)_. Given noisy data and nonunique kmers, however, no single match is guaranteed to be correct. Furthermore, due to potential insertion and deletion errors, all correct matches may still not agree on a precise alignment. Therefore, we search for the alignment that (roughly) explains the maximum number of kmer matches as follows. First, we let each kmer in the read matching a kmer in the contig vote for the respective alignment positions. As a result we obtain a vector **V** of votes for each position in the contig. Since we expect some votes to be split across neighbouring positions due to indels, we perform a simple convolution over **V** to collect neighbouring votes, resulting in a new vector 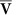, where 
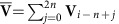
 (we use *n*=10 in our experiments). The position in 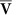 with the maximum number of votes is selected as the best alignment position, with the number of votes representing its score (if a range of positions have the maximum number of votes, we pick the middle position in the range). As we process all the contigs, we keep the best score *r*_1_ and second best score *r*_2_ found per read to identify situations when a read can map to multiple positions and estimate the mapping quality of our alignments. We currently use the simple formula α(*r*_1_−*r*_2_)/*r*_1_ (similar to other methods like BWA-SW^3^) for our mapping quality. We do not reveal the kmer positions in the reference genome during voting, since only relative positions are needed to compute the displacement and count the votes. The final reported alignment position for each confidently mapped read is computed on the client by adding the global offset of the contig with the highest score to the best local displacement found for this contig.

To minimize bandwidth, we only send a fraction of contig kmers to the cloud to participate in voting. The configurable sampling rate parameter, *ρ*, controls the sparsity of the sampled kmers.

### Threat assumptions

We assume the ‘honest-but-curious' adversary cloud model that follows the read alignment protocol correctly but will try to infer the read sequences from the input data it sees and any background information it has obtained. The computation on the private client and the client–server communications are assumed to be secure. Our goal is to prevent the cloud from deciphering the original read sequences that can lead to the identification of the individual they came from.

We reasonably assume that the adversary has access to the standard reference genome used during alignment. This knowledge enables numerous statistical attacks if repeats can be detected in the data. We differentiate between LRS, formed by equal kmers inside each read or contig, and GRS, formed by equal kmers across sequences. The information leaked by both types of repeat structures can be exploited in a variety of attacks.

For instance, an LRS attack counting kmers can be designed as follows. For each window of the genome (for example, of length equal to read length), the adversary can precompute the kmer histogram representing how many times different kmers occurred in this window. Using LRS, similar histograms can also be computed for each read. Matching read and reference histograms can then potentially uncover the read sequences. We instrumented such an attack to assess how identifiable are the reference windows from their kmer histograms. For a window length of 150 bp, we found 91% of the windows of the genome to have no 20 bp long repeat kmers (that is, every kmer occurs exactly once, making such windows unidentifiable); however, various unique patterns emerged in the remaining 9% of the windows, suggesting that leaking LRS is not secure.

Similarly, the GRS information can be exploited by counting how many times each kmer occurred across the entire read data set. Assuming that this frequency is equivalent to the kmer frequency inside the reference genome, the adversary can create a mapping between plaintext sequences and hashed kmers found to occur with similar frequencies (this attack was analysed by Chen *et al*.^14^ since their algorithm allows the adversary to compute such frequencies; however, they determined that this information would not offer much re-identification power to the attacker). GRS information can be further exploited if the relative positions of the kmers inside each sequence are also known. For instance, the adversary can attempt to assemble reads or initial contigs into longer contigs by detecting suffix–prefix kmer overlaps (for example, using a standard overlap graph assembly algorithm). Longer contigs can then enable more effective histogram attacks since their kmer histograms would result in signatures that are more unique (especially when coupled with kmer position information). Furthermore, identifying repeating kmer tuples in non-overlapping sequences can also leak information due to potential unique co-occurrence patterns (we refer to attacks using this information as GRS-pattern attacks). For example, if two kmers are observed in one contig at a relative distance *x* but in a different contig at a relative distance *y*, then if there exists a single pair of kmers that co-occur at distances *x* and *y* in the reference genome, we can exactly identify these two kmers. Any number of more complex co-occurrence patterns can be similarly exploited.

Given the above vulnerabilities, our voting scheme is designed not to leak LRS and GRS information, as described below.

### Secure kmer voting

A key property of our voting protocol is that it does not require the comparison of kmers across voting tasks. This allows us to avoid leaking GRS information by associating unique secret keys with each voting task.

We compute the kmer keyed hash values as follows. First, we apply a standard cryptographic hash function *h*_*K*_ to each kmer. We use SHA-1 with a 256-bit secret key *K*; our kmer hash consists of the first 64 bits of its digest (note that both SHA-1 and the length of the kmer hash can be replaced in our method by another cryptographic hashing scheme without affecting its accuracy). To prevent GRS, we can just select *K* to be unique per task. However, to optimize the hashing runtime, we use the same key *K* for all the tasks, allowing us to precompute the *h*_*K*_ values of the reference kmers before mapping, and then quickly modify each digest using a set of two randomly generated 64-bit keys, (*k*_1_^*T*^, *k*_2_^*T*^), unique for each voting task *T*. Namely, the hash of kmer *s*_*i*_ is set to: ((*h*_*K*_(*s*_i_) ⊕ *k*_1_^*T*^) × *k*_2_^*T*^). As a result, kmer collisions can be detected within each task but not across different tasks.

Next, to avoid LRS leaks, we mask repeat kmers inside each read and contig (that is, replace their hash values with a random 64-bit integer). However, such masking can be detected when evaluating the matching pattern between multiple kmers. For example, if we observe a kmer mismatch enclosed by kmer matches in both the read and the contig, we can deduce that the mismatch is due to masking (since the matching predecessor and successor kmers will entirely cover the bases of the mismatching kmer). To avoid this leak, we mask the neighbouring kmers of each repeat as well, such that there are at least *v* masked kmers around each repeat, where *v* is the length of the voting kmers.

To mediate the effect of masking repeat neighbourhoods on alignment accuracy (see Supplementary Fig. 1), as well as reveal less information about the pattern with which the read kmers matched the contig, our protocol discloses only the partial ordering of the kmers inside the sequence. More specifically, the kmers are broken down into bins of size *β*, such that *i*th bin *B*_*i*_ contains *β* kmers in an arbitrary random order that fall in the range [*l*, *h*)=[*i* × *β*, *i* × *β*+*β*) (for example, *β*=1 would reveal the full ordering of the kmers). In our experiments, we use a bin size of *β=*20 (equal to the length of the kmers).

**Figure 5:**
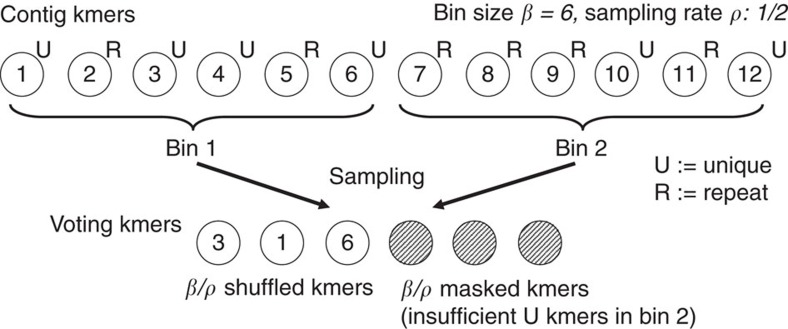
Selection of voting kmers. Illustration of the procedure applied to contig kmers during voting in phase 2.

With binning, each kmer match casts a vote for a range of positions (as opposed to a single position) equivalent to the positions obtained if every single kmer in the contig bin matched every kmer in the corresponding read bin, namely: [*l*^*C*^−*h*^*R*^+1, *h*^*C*^−*l*^*R*^]. When sampling is enabled, the number of kmers expected per bin must account for the sampling rate, *ρ*. If there are *β*/*ρ* unique kmers in the bin, all these kmers can be selected for voting and the bin will not be masked even when repeats are present; otherwise, masking must be applied on the entire bin. Figure 5 illustrates the binning, sampling and masking steps applied when selecting the kmers sent for voting.

In the end, the main information the adversary has is the result of each voting task: the number of kmers matched and their binned local positions. Additionally, we also reveal the number of kmers sent for each candidate contig. However, the contig length can be easily hidden by splitting long contigs into multiple chunks (each assigned to a separate voting tasks) and padding the lengths with randomly generated kmers. These operations do not affect alignment accuracy, while having a negligible compute and communication overhead (since the amount of padding used and the number of required splits can be expected to be small). We are unaware of any statistical attacks that could use the information seen by the cloud; however, we do not prove categorically that an attack will not be discovered in the future.

### Code availability

Balaur is free and open-source software implemented in C++ and available at http://viq854.github.com/balaur.

### Data availability

All relevant data are available from the corresponding authors.

## Additional information

**How to cite this article:** Popic, V. & Batzoglou, S. A hybrid cloud read aligner based on MinHash and kmer voting that preserves privacy. *Nat. Commun.* **8,** 15311 doi: 10.1038/ncomms15311 (2017).

**Publisher's note**: Springer Nature remains neutral with regard to jurisdictional claims in published maps and institutional affiliations.

## Supplementary Material

Supplementary Information Supplementary Figures, Supplementary Tables, Supplementary Notes and Supplementary References
