# Supplementary material for: A hybrid cloud read aligner based on MinHash and kmer voting that preserves privacy
Source: Nat Commun. 2017 May 16;8:15311. doi: 10.1038/ncomms15311 (PMC5440850; doi:10.1038/ncomms15311)
Supplement: Supplementary Information — Supplementary Figures, Supplementary Tables, Supplementary Notes and Supplementary References [file ncomms15311-s1.pdf]

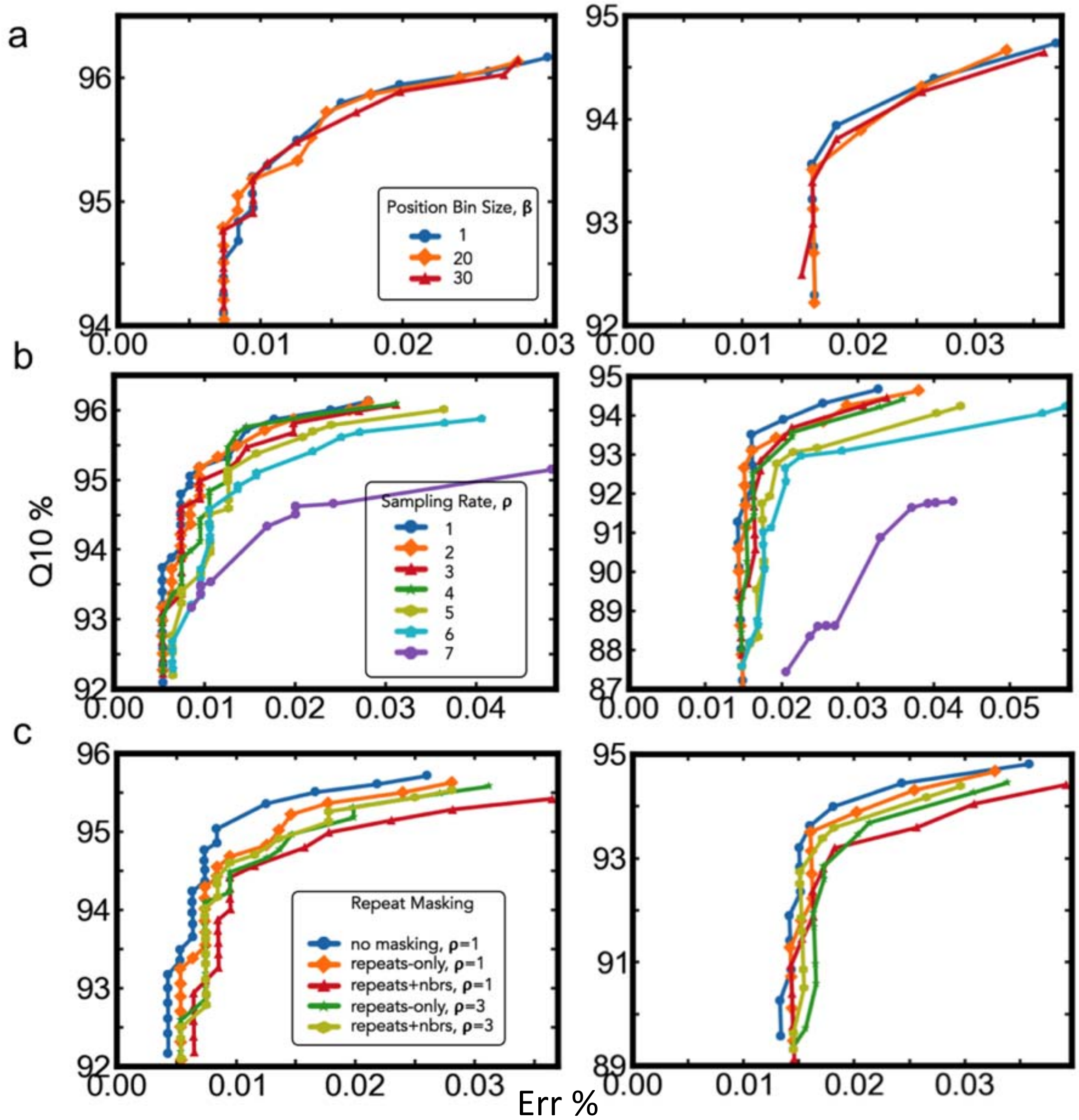

Supplementary Figure 1. *Evaluation of the effects of kmer position binning, sampling, and LRS masking on alignment accuracy of 150-bp read datasets simulated from GRCh37 with wgsim[1] using default parameters and sequencing error rates of 1% (left) and 2% (right).* (a) The bin size parameter, β, controls the ambiguity in the kmer positions inside the read or contig, such that each kmer is ambiguous within β positions and only the relative positions of the bins are known (e.g. $\beta = 1$ would reveal the full ordering of the kmers). All experiments were run without sampling ($\rho = 1$) and LRS masking ’repeat-only’ mode. (b) The sampling rate parameter, ρ, controls the sparsity of the contig kmers sent for voting to the cloud: $1/\rho$ fraction of the kmers are used from each bin. All experiments were run with bin size $\beta = 20$ and LRS masking ’repeat-only’ mode. (c) LRS masking ’repeats-only’ mode masks only the kmers occurring more than once in the read or contig; ’repeats+nbrs’ mode masks the repeat kmers and their neighboring kmers, such that for dense read kmers, each masked repeat is surrounded by at least v masked kmers (where v is the kmer length) and for sparse contig kmers, each kmer bin is masked entirely if its fraction of unique kmers is less than $1/\rho$, otherwise the unique kmers are shipped instead. All experiments were run with bin size $\beta = 20$.

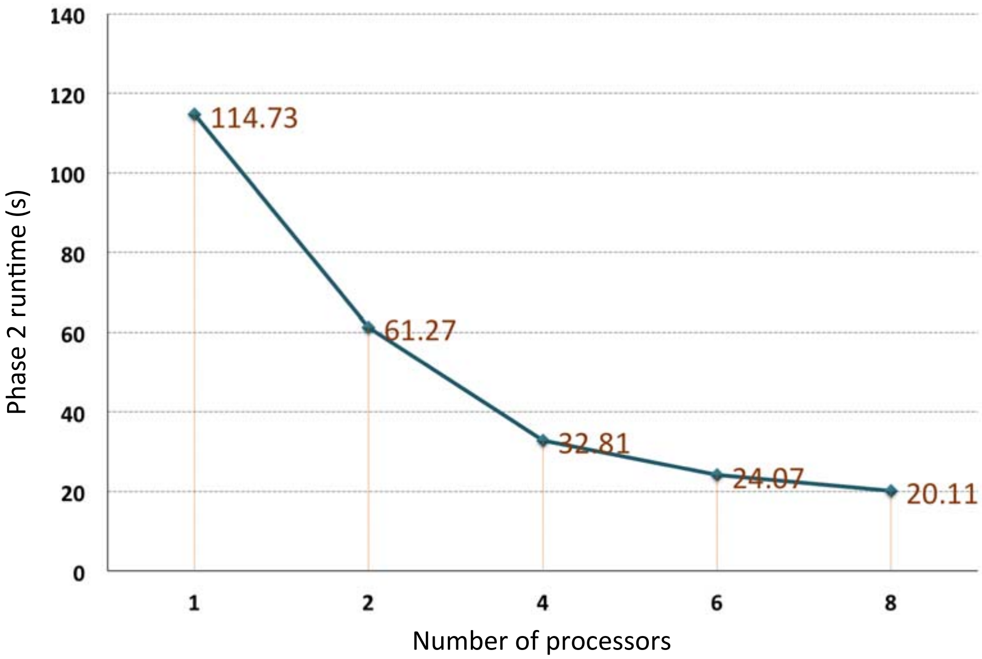

Supplementary Figure 2. *Evaluation of the scalability of the Phase 2 voting computation with respect to the number of parallel processors.* Results are shown for 500K real 150-bp HiSeq2500 reads of the NA12878 genome.

Length	Program	1%			2%		
		Q10%	Err%	Time (s)	Q10%	Err%	Time (s)
150-bp	Balaur	96.0	0.03	68	94.4	0.03	95
	Bowtie2	95.9	0.02	68	94.5	0.03	65
	BWA-MEM	96.8	0.01	57	96.8	0.02	73
	ALFALFA	96.9	0.03	51	97.1	0.2	46
	CUSHAW3	96.8	0.002	82	96.7	0.02	91
	SOAP2	95.8	0.06	44	92.5	0.1	82
350-bp	Balaur	97.8	0.004	79	96.8	0.005	120
	Bowtie2	97.4	0.006	178	96.5	0.001	169
	BWA-MEM	98.4	0.002	169	98.3	0.002	192
	ALFALFA	98.0	0	88	97.9	0	86
	CUSHAW3	97.7	0	401	97.4	0	415
	SOAP2	94.4	0.03	143	90.9	0.02	168

Supplementary Table 1. *Performance evaluation on simulated data.* Each dataset consists of 100,000 reads.

Dataset	Program	Q10%	Time (s)
NA12878	Balaur	88.4	416
	Bowtie2	88.8	808
	BWA-MEM	91.2	614
	ALFALFA	93.1	587
	CUSHAW3	92.3	611
	SOAP2	87.9	917
NA24385	Balaur	87.6	456
	Bowtie2	88.0	693
	BWA-MEM	90.8	530
	ALFALFA	92.7	569
	CUSHAW3	92.1	658
	SOAP2	87.2	995

Supplementary Table 2. *Evaluation on real data*. Each dataset consists of 1M reads.

Pipeline	True+	False+	True-	False-
Balaur + GATK_HC	8,899	17,804	37,220,787	6,451
Bowtie2 + GATK_HC	7,148	14,929	37,223,662	8,175
BWA + GATK_HC	1,885	548	37,238,043	13,715
Novoalign + GATK_HC	6,785	12,250	37,226,341	8,620

Supplementary Table 3. *Evaluation of variant calling using the GCAT benchmarking platform.* Results are shown for the NA12878 Ion Torrent 225-bp SE exome 30x dataset available through GCAT. Results in the table were generated by GCAT in comparison against the NIST Genome in a Bottle SNP and indel call set (v2.18). For more information please see Supplementary Note 2 and the caption of Fig. 4.

Error, Len	Program	Q10%	Err%	Time (s)	Speedup
	Balaur-v (1)	99.2	0	69	
	Balaur-v (2)	99.2	0	80	
1%, 1K	BWA-MEM	99.2	0	495	7.2/6.2
	Bowtie2	97.9	0.002	2,781	40.3/34.8
	BWA-SW	98.3	0.002	1,026	14.9/12.8
	ALFALFA	98.3	0	169	2.4/2.1
	Balaur-v (1)	99.1	0.020	71	
	Balaur-v (2)	99.1	0.008	106	
2%, 1K	BWA-MEM	99.1	0	702	9.9/6.6
	Bowtie2	97.4	0.002	2,659	37.5/25.1
	BWA-SW	98.2	0.004	948	13.4/8.9
	ALFALFA	98.3	0	230	3.2/2.2
	Balaur-v (1)	99.4	0.003	471	
	Balaur-v (2)	99.5	0	555	
4%, 10K	BWA-MEM	99.9	0	7,781	16.5/14.0
	BWA-SW	99.2	0.002	6,094	12.9/11.0
	ALFALFA	98.8	0.02	1,621	3.4/2.9
	Balaur-v (3)	99.4	0.003	677	
	Balaur-v (4)	99.4	0.004	1,132	
8%, 10K	BWA-MEM	99.8	0	7,507	11.1/6.6
	BWA-SW	99.2	0	5,284	7.8/4.7
	ALFALFA	98.8	0.180	2,806	4.1/2.5
	Balaur-v (5)	99.4	0.015	1,311	
10%, 10K	BWA-MEM	99.8	0	7,024	5.4
	BWA-SW	99.2	0.001	5,056	3.9
	ALFALFA	98.2	0.175	4,495	3.4

Supplementary Table 4. *Evaluation on simulated long read datasets.* Each dataset consists of 100,000 reads. The results were obtained using three different MHG indices, constructed with the following parameters. MHG0: L=128, T=78, b=2, M=18 (index size 1.1GB); MHG1: L=256, T=512, b=2, M=16 (index size 6.7GB); MHG2: L=256, T=1024, b=2, M=16 (index size 14GB). The index used in each run is indicated by the setting numbers (1)-(5), where: (1) MHG0, $b_m = 2$, (2) MHG0, $b_m = 1$, (3) MHG2, $b_m = 4$, (4) MHG1, $b_m = 1$; (5) MHG2, $b_m = 1$, where b_m stands for b_{min_hits}. Kmer sampling rate, ρ, was also set as follows: $\rho = 4$ (1K); $\rho = 8$ (10K, 4%); $\rho = 2$ (10K, 8% and 10 %).

Length	Phase 1 Runtime (s)		Phase 2 Runtime (s)			Total Time (s)	Bandwidth	
	MinHash	Merge	Encryption	Voting	Reporting		Up (MB)	Down (MB)
150-bp, 1%	2.9	7.4	10.3	45.8	2.0	68.4	4,930	110
150-bp, 2%	2.9	7.0	14.3	68.8	2.0	95	7,507	168
350-bp, 1%	7.0	5.6	12.7	49.4	4.4	79.1	4,651	44
350-bp, 2%	7.0	5.4	17.2	86.1	4.4	120.1	8,323	80
NA12878	27.7	76.4	64.3	227.0	20.3	415.7	24,613	554
NA24385	28.7	75.0	70.6	260.0	20.5	455.8	28,423	641

Supplementary Table 5. *Runtime and bandwidth breakdown by alignment phase.*

Supplementary Note 1: Alignment evaluation experimental setup

Datasets

To evaluate the accuracy of our aligner, we simulated reads from the full human genome reference GRCh37 using the wgsim [1] program. For short read experiments, we created datasets of 150-bp and 350-bp long reads with default parameters (0.09% SNP mutation rate and 0.01% indel mutation rate) and sequencing base error rates of 1% and 2%. For long read experiments, we created datasets of 1K-bp and 10K-bp long reads with default polymorphism parameters and the base error rates of: 1% and 2% (for 1K-bp) and 4%, 8%, and 10% (for 10K-bp). Each simulated dataset included 100,000 reads.

We used the following two datasets in our real read experiments: (1) 150-bp HiSeq2500 reads of the NA12878/HG001 genome and (2) 150-bp reads of the NA24385/HG002 genome. The datasets were obtained from the NIST Genome in a Bottle repository and were subsampled to 1M reads each.

Balaur (v0.0.1)

MHG Construction. Across all experiments, the MHG index was constructed using 16-bp kmers (-k 16), a kmer maximum occurrence cutoff of 800 (-H 800, i.e. kmers occurring in the genome more than 800 times were excluded from the MinHash fingerprint computation), and fingerprint projections of length 2 (-b 2). The length of the indexed reference windows was set according to the read length used in each experiment. The length of the MinHash fingerprints, the number of hash tables, and the number of per table buckets varied as described below.

Short read experiments. For real reads and simulated datasets of 150-bp and 350-bp reads, Balaur results were obtained with MHG parameters: L = 128 (fingerprint length), T = 78 (number of hash tables), M = 18 ($2M$ per table buckets). We used a voting kmer length of 20-bp, contig kmer sampling rate of ρ = 3, position bin size β = 20, convolution radius of 10, the LRS neighbor masking mode, and no contig batching in voting tasks. These settings correspond to the default short read program options: -h 128 -T 78 -p 18 -v 20 -I 3 -B 20 -d 10 -M -S 1. For candidate contig filtering we also used the default setting of 1 for the $b_{\text{min\_hits}}$ parameter (-m 1), which represents the minimum number of MHG buckets that must contain the contig for it to be selected as a candidate (this parameter is dynamically increased to 2 if the number of candidate contigs found per read exceeds 10K). Furthermore, for 1% error experiments we used (-N 25) to relax candidate contig filtering

based on how many buckets contained the contig w.r.t. the highest number of buckets observed for any candidate contig of the given read, while for the real reads and 2% error experiments we used the default options -N 20. The non-secure version of our method, Balaur-vanilla (-V mode), used a faster non-cryptographic hash function (namely, CityHash64), no LRS masking, and no position binning. We did not incorporate initialization costs (e.g. the allocation of kmer transfer buffers and MHG loading) into the reported Balaur runtime.

Long read experiments. These experiments used the Balaur-vanilla implementation, voting kmer length of 32-bp (-v 32), and varying kmer sampling rates and MHG index sizes (the specific configurations are presented in Supplementary Table 4). The results shown in Fig. 3d correspond to the configurations of Supplementary Table 4 that maximized the number of correct Q10 mappings.

ALFALFA (v0.8.1)
According to authors' suggestion in the ALFALFA manuscript [2], global alignment mode was chosen for short reads (< 500-bp) and local alignment was enabled for longer reads using the --local flag. Long read experiments were executed in two modes: (1) using defaults and (2) setting the maximum edit distance parameter -e to the wgsim simulation error rate used for the dataset (namely, 0.04, 0.08, and 0.1). We report the results obtained in mode (2) since they showed improvements over the default settings. In all experiments we used the default setting -a 1, which reports one alignment per read.

Commands:
alfalfa index -r <ref.fa>
alfalfa align -i <ref.index> -0 <reads.fq> -o <aln.sam>
alfalfa align -i <ref.index> -0 <reads.fq> -o <aln.sam> [-e <wgsim error>] --local

Bowtie2 (v2.2.6)
We evaluated Bowtie2 [3] with default parameters.

Commands:
bowtie2 index <ref.fa> <index prefix>
bowtie2 -x <index prefix> -U <reads.fq> -S <aln.sam>

BWA-MEM [4] and BWA-SW [5] (v0.7.12)
We evaluated both programs with their default parameters across all datasets using the same index.

Commands:
bwa index <ref.fa> bwa [mem | sw] <ref.fa> <reads.fq> > <aln.sam>

CUSHAW3 (v3.0.3)
We evaluated CUSHAW3 [6] with default parameters and the -multi 1 setting.

Commands:
cushaw3 index <ref.fa>
cushaw3 align -r <ref.fa> -f <reads.fq> -o <aln.sam> [-multi 1]

SOAP2 (v2.21)

SOAP2 [7] was evaluated with seed length -l 28 and maximum number of mismatches -v 7 (for 150-bp reads) and 12 (for 350-bp reads). The parameter -v was set according to the given read length and error rate of 0.02. We found parameter -g to have no effect. All other parameters were left as defaults. All the experiments were also performed with default parameters to ensure our setting results in better performance.

Commands:

```
2bwt-builder <ref.fa>
soap -D <ref.index> -a <reads.fq> -o <aln.sam> -l 28 -v [7, 12]
```

Supplementary Note 2: Variant calling experimental setup

We obtained the NA12878 Ion Torrent 225-bp 30x exome read dataset from the GCAT benchmarking platform [8]. We used GATK HaplotypeCaller (v3.6), Picard tools (v1.92), and Samtools (v1.3.1) in our variant calling pipeline for this dataset, closely following the GATK Best Practices guidelines [9] with a few non-default settings. Starting with our alignment results in SAM format balaur.sam, we applied the commands below. The final VCF file was uploaded to GCAT for analysis and comparison with other tools.

Our variant calling pipeline commands:

```
samtools view -S -b -T ucsc.hg19.fasta balaur.sam > balaur.bam

PICARD/CleanSam.jar I=balaur.bam O=balaur clean.bam

PICARD/AddOrReplaceReadGroups.jar I=balaur clean.bam O=balaur temp.bam RGID=4
RGLB=lib1 RGPL=ion RGPU=unit1 RGSM=20

PICARD/SortSam.jar SO=coordinate INPUT=balaur temp.bam OUTPUT=balaur
sorted.bam VALIDATION STRINGENCY=LENIENT CREATE INDEX=true

GenomeAnalysisTK.jar -T RealignerTargetCreator -R ucsc.hg19.fasta -I -I balaur
sorted.bam -o target intervals.list --windowSize 500 --minReadsAtLocus 1 --
mismatchFraction 0.5 -nt 10

GenomeAnalysisTK.jar -T IndelRealigner -R ucsc.hg19.fasta -I balaur sorted.bam
-o balaur sorted rln.bam -targetIntervals target intervals.list -model USE SW

GenomeAnalysisTK.jar -T HaplotypeCaller -R ucsc.hg19.fasta -I balaur sorted
rln.bam -o balaur.vcf -minPruning 5 -nct 20
```

Supplementary Note 3: Balaur parameter description

Below is a summary of user-controlled Balaur parameters. MinHash and MHG index parameters:

k: kmer length used when converting reference windows or read sequences to sets of overlapping kmers for MinHash fingerprinting (16)

L: number of hash functions used for MinHash fingerprint construction (i.e. fingerprint length)

T: number of hash tables in the MHG index (equal to the number of b-dimensional sparse projections computed from the fingerprint)

b: length of the fingerprint projections

B: the number of buckets in each MHG hash table (s.t. $B = 2M$)

Alignment and privacy parameters:

$b_{\text{min\_hits}}$: minimum number of MHG index buckets shared with the read required for a contig to be selected as a candidate

$b_{\text{best\_hits}}$: highest number of bucket hits shared between a read and any contig

N: maximum distance from b_{best} hits for a contig to be selected as a candidate

v: kmer length used during the voting step (20)

n: votes array convolution radius (10)

β: voting kmer discretized position range (bin size), within which the kmer position is ambiguous (20)

ρ: voting kmer sampling rate (used to reduce the bandwidth and for LRS masking)

S: voting task size (or batch size): number of contigs per read hashed with same keys (safest setting is 1; however, a larger value will reduce the bandwidth)

Supplementary Note 4: Background on locality sensitive hashing and the MinHash algorithm

Locality sensitive hashing (LSH) is a probabilistic dimensionality-reduction technique that has been introduced by Indyk and Motwani [10] to address the approximate similarity search problem in high dimensions. Its key property is to maximize the probability of collision of objects that are similar. In particular, let H be a family of hash functions h: $R^d \rightarrow U$. An LSH scheme defines a probability distribution over the family H, such that given two objects $x, y \in R^d$: $Pr_{h \in \mathrm{H}}[h(x) = h(y)] = similarity(x, y)$.

For example, a simple family of functions H can be constructed for d-dimensional binary vectors from $\{0, 1\}^d$ under the Hamming distance metric. In this case, the family of functions can just consist of all the projections of the input points from $\{0,1\}^d$ onto one of the d vector coordinates; namely, of the functions $h_i(x) = x_i$, where $i \in \{1,...,d\}$ is a random index into the vector x. It can be easily seen that under such hash functions, the probability of collision of the hashes of two given vectors will be equal to the fraction of coordinates that are equal between the two vectors. For a survey of different LSH families see [11]. In computational biology, LSH has been applied to several tasks, including motif discovery [12], genome-wide association studies [13], and recently SMS read overlap detection for de novo assembly [14].

In this work we apply the MinHash LSH scheme to hash the reference genome and the reads, such that the hash values (referred to as *fingerprints*) of the read and its genome window collide with high probability. Since the read can differ from the reference sequence it maps to due to sequencing errors and true genomic variants, our similarity measure needs to handle differences in the two sequences arising from base substitutions and indels. A standard approach for measuring the similarity between two strings is to represent them as sets (e.g., a set of all the words in a document). Then the similarity of two such sets A and B can be expressed by their *Jaccard coefficient*: $J(A,B) = |A \cap B| / |A \cup B|$. Several LSH families have been proposed for the Jaccard similarity criterion. Below we describe one of the most popular such techniques; namely, the MinHash algorithm, which we applied in this work.

The *min-wise independent permutations* (MinHash) LSH family has been proposed by Broder et. at [15] for the Jaccard similarity measure and is defined as follows. Let U be the ground

set of all possible set items. Given a random permutation π of indices of U and a set X, let $h_\pi(X) = \min_{x \in X}\{\pi(x)\}$. The MinHash LSH family H will consist of all such functions for each choice of π. It can be easily shown that for a given h chosen uniformly at random, $\Pr[h_\pi(A) = h_\pi(B)] = J(A,B)$ (see [15] for details). Due to the high variance in the probability of collision, an amplification process is usually applied. More specifically, instead of using one hash function, we concatenate L different hash functions from the family H chosen independently at random. It can be shown that given the number of the hash collisions among the chosen L functions, c, the ratio c/L can also be used as an unbiased estimator for $J(A,B)$. Since computing random permutations can be prohibitive, the hash functions are typically created using universal hash functions of the form: $h(x) = ax + b$. We follow a similar approach in our method.

Supplementary Note 5: Rolling MinHash fingerprinting

Using the fact that consecutive windows of the genome are related, we developed a rolling MinHash technique to more efficiently compute the fingerprint of window w_{p+1} from that of window w_p, where p and $p + 1$ are consecutive positions in the genome. This technique is applied as follows.

Let M be a matrix of size $L \times n$, where L is the length of the fingerprint and n is the size of the kmer set. The function MinHashRoll shown in Algorithm 1 takes as argument the matrix M, the column index m_{oldest}, and the fingerprint vector F_w obtained for window w_p; it then updates the state of these three variables accordingly, with the fingerprint of w_{p+1} being stored in F_w. The three variables are initialized for the first genome window w_0 as follows: $M(i,j) = h_i(w_{0j})$, where w_{0j} is the kmer at position j in w_0, $m_{oldest} = 0$, and $F_w(i) = \min(M(i,\cdot))$. The MinHashRoll procedure is then applied for all successive windows $p > 0$. The optimization is due to the fact that the minimum over each row of M only needs to be recomputed when the w_p minimum value in that row comes from a column corresponding to m_{oldest} and is smaller than the hash value of the last kmer in w_{p+1}. This technique greatly reduced the computation time of the indexing step.

Algorithm 1 Rolling MinHash

procedure MinHashRoll(M, m_{oldest}, F_w)
$w_{p+1,last} \leftarrow$ last kmer in window w_{p+1}
$H_{last} \leftarrow H(w_{p+1,last})$ // kmer hash
for i=0 **to** L **do**
  $\min_h \leftarrow h_i(Hl_{ast})$
  if $\min_h < F_w(i)$ **then**
    $F_w(i) \leftarrow \min_h$
    $M(i, m_{oldest}) \leftarrow \min_h$
  else if $M(i, m_{oldest})$!= $F_w(i)$ **then**
    $M(i, m_{oldest}) \leftarrow \min_h$
  else
    $F_w(i) \leftarrow$ MAX VAL
    $M(i, m_{oldest}) \leftarrow \min_h$
    for j = 0 **to** n **do**
      if $M(i,j) < F_w(i)$ **then**
        $F_w(i) \leftarrow M(i,j)$
$m_{oldest} \leftarrow (m_{oldest} + 1) \bmod n$

Supplementary References

[1] Li, H.: wgsim-read simulator for next generation sequencing (2011)

[2] Vyverman, M., De Baets, B., Fack, V., Dawyndt, P. A long fragment aligner called alfalfa. BMC bioinformatics 16(1), 1 (2015).

[3] Langmead, B., Salzberg, S.L. Fast gapped-read alignment with bowtie 2. Nature methods 9(4), 357–359 (2012).

[4] Li, H. Aligning sequence reads, clone sequences and assembly contigs with bwa-mem. arXiv preprint arXiv:1303.3997 (2013).

[5] Li, H., Durbin, R. Fast and accurate long-read alignment with burrows–wheeler transform. Bioinformatics 26(5), 589–595 (2010).

[6] Liu, Y., Popp, B., Schmidt, B. Cushaw3: sensitive and accurate base-space and color-space short-read alignment with hybrid seeding. PloS one 9(1), e86869 (2014).

[7] Li, R., Yu, C., Li, Y., Lam, T.W., Yiu, S.M., Kristiansen, K., Wang, J. Soap2: an improved ultrafast tool for short read alignment. Bioinformatics 25(15), 1966–1967 (2009).

[8] Highnam, G., Wang, J.J., Kusler, D., Zook, J., Vijayan, V., Leibovich, N., Mittelman, D. An analytical framework for optimizing variant discovery from personal genomes. Nature communications 6 (2015).

[9] DePristo, M.A., Banks, E., Poplin, R., Garimella, K.V., Maguire, J.R., Hartl, C., Philippakis, A.A., Del Angel, G., Rivas, M.A., Hanna, M., et al. A framework for variation discovery and genotyping using next-generation dna sequencing data. Nature genetics 43(5), 491–498 (2011).

[10] Indyk, P., Motwani, R. Approximate nearest neighbors: towards removing the curse of dimensionality. Proceedings of the thirtieth annual ACM symposium on Theory of computing, 604–613 (1998).

[11] Andoni, A., Indyk, P. Near-optimal hashing algorithms for approximate nearest neighbor in high dimensions. 47th Annual IEEE Symposium on Foundations of Computer Science, 459–468 (2006).

[12] Wijaya, E., Rajaraman, K., Brahmachary, M., Bajic, V., Yuan, S. A hybrid algorithm for motif discovery from dna sequences. 3rd Asia-Pacific Bioinformatics Conference (2005).

[13] Brinza, D., Schultz, M., Tesler, G., Bafna, V. Rapid detection of gene–gene interactions in genome-wide association studies. Bioinformatics 26(22), 2856–2862 (2010).

[14] Berlin, K., Koren, S., Chin, C.S., Drake, J., Landolin, J.M., Phillippy, A.M. Assembling large genomes with single-molecule sequencing and locality sensitive hashing. bioRxiv p. 008003 (2014).

[15] Broder, A.Z., Charikar, M., Frieze, A.M., Mitzenmacher, M. Min-wise independent permutations. Journal of Computer and System Sciences 60(3), 630–659 (2000).
